# Large, persistent epidemic of adenovirus type 4-associated acute respiratory disease in U.S. army trainees.

**DOI:** 10.3201/eid0506.990609

**Published:** 1999

**Authors:** R M Hendrix, J L Lindner, F R Benton, S C Monteith, M A Tuchscherer, G C Gray, J C Gaydos

**Affiliations:** Dwight David Eisenhower Army Medical Center, Fort Gordon, Georgia, USA. mcneilkm@ncstr60.dhec.state.sc.us

## Abstract

In May 1997, a large, persistent epidemic of adenovirus type 4-associated acute respiratory disease began at Fort Jackson, South Carolina, the largest army basic training center. The epidemic lasted until December and declined when vaccine administration resumed. More than 1,000 male and female trainees were hospitalized; 66.1% of those hospitalized had an adenovirus type 4 isolate.


### Large, Persistent Epidemic of Adenovirus Type 4-Associated Acute Respiratory Disease in U.S. Army Trainees

**K. Mills McNeill,* Rose M. Hendrix,† Jane L. Lindner,‡ F. Ridgely Benton,* Susan C. Monteith,* Margaret A. Tuchscherer,* Gregory C. Gray,§ and Joel C. Gaydos¶**

*Dwight David Eisenhower Army Medical Center, Fort Gordon, Georgia, USA; †University of South Carolina School of Medicine, Columbia, South Carolina, USA; ‡Moncrief Army Community Hospital, Fort Jackson, South Carolina, USA; §Naval Health Research Center, San Diego, California, USA; ¶Department of Defense Global Emerging Infections System, Washington, D.C., USA

In May 1997, a large, persistent epidemic of adenovirus type 4-associated acute respiratory disease began at Fort Jackson, South Carolina, the largest army basic training center. The epidemic lasted until December and declined when vaccine administration resumed. More than 1,000 male and female trainees were hospitalized; 66.1% of those hospitalized had an adenovirus type 4 isolate.

Nonvaccine interventions have proven unreliable in the control of adenovirus-associated acute respiratory disease (1). Before live, oral, enteric-coated adenovirus types 4 and 7 vaccines were introduced in 1971, adenovirus-associated acute respiratory disease produced high attack rates and excessive illness in soldiers during service-entry basic combat training (2). The subsequent policy to vaccinate all male trainees during basic combat training drastically reduced adenovirus type 4- and type 7-associated acute respiratory disease (3).

In 1996, the sole manufacturer of the vaccines ceased production (4). To conserve the remaining vaccine lots, the army restricted use of adenovirus vaccines to the period of September 1 through March 31, the peak season for acute respiratory disease. Administration of adenovirus vaccines to military trainees was suspended on March 31, 1997, at all army basic training centers. To monitor the impact of this modified policy, intensive, laboratory-based surveillance was initiated in late April 1997 at Fort Jackson, South Carolina, the army's largest basic combat training center. An epidemic of adenovirus type 4-associated acute respiratory disease occurred among male and female soldiers in basic combat training at Fort Jackson after the vaccination was suspended. The first case appeared in late May, and the outbreak lasted until December, after vaccination was resumed in November.

Address for correspondence: K. Mills McNeill, Catawba Health District, P.O. Box 817, Lancaster, SC 29721, USA; fax: 803-286-5418; e-mail: mcneilkm@lncstr60.dhec.state.sc.us.

### The Outbreak

During May through December 1997, a monthly average of 6,847 soldiers from all geographic regions of the United States and its protectorates were engaged in the 8-week basic combat training program at Fort Jackson. During this period, 38.2% of the trainees were women. The mean age of soldiers enrolled in this study was 19.7 years.

All trainees who report to sick call with fever of 100.5° F or higher are admitted to a minimal-care hospital ward at the Fort Jackson Army Hospital for observation and self-care. Soldiers from this group who had an oral temperature of 100.5° F or higher, plus at least one sign or symptom of an upper respiratory infection, were enrolled in the surveillance program. Approximately 80% of soldiers hospitalized with acute respiratory disease were enrolled since patients admitted on weekends were not included. A pharyngeal swab was taken from each trainee and immediately placed into virus transport medium (Viromed Laboratories, Inc., Minneapolis, MN). Specimens were stored at 2°C to 8°C for an average of 72 hours before overnight express shipment on wet ice to the laboratory for virus isolation.

Virus isolations and identifications were performed by the Department of Pathology Laboratory at Dwight David Eisenhower Army Medical Center. Adenoviruses were isolated in human lung carcinoma (A-549) cells (Viromed Laboratories, Inc., Minneapolis, MN). The serotype of isolated adenoviruses was determined by virus neutralization with type-specific antisera (Centers for Disease Control and Prevention, Atlanta, GA); the Reed-Muench method was used for calculating the $LD_{50}$ titer (5). Each culture was also tested for influenza A and B; parainfluenza 1, 2, and 3; herpes simplex virus; and enteroviruses by cell culture, enzyme immunoassay, and fluorescent antibody staining.

The first isolate of adenovirus type 4 came from a patient who was hospitalized on May 22, 1997, approximately 7 weeks after administration of the adenovirus vaccines ended. During May through December 1997, 1,018 basic trainees whose illness met the case definition were examined. Of these, 673 (66.1%) were positive for adenovirus type 4. No other respiratory disease agent was identified as an important cause of illness in this epidemic. The monthly case distribution was calculated by sex for all hospitalized acute respiratory disease patients (Figure 1) and for adenovirus type 4-positive patients (Figure 2). Of the total acute respiratory disease cases, 35.3% (only slightly less than their representation in the total trainee population of 38.2%) were in women. Similarly, 31.2% of all adenovirus type 4 isolates were from women. During May, June, and July, the monthly rates of acute respiratory disease admissions were higher for women than for men (Figure 1). These higher rates were not due to adenovirus type 4 (Figure 2) or any other agent tested for in this study.

The percentage of cases from which adenovirus type 4 was isolated increased as the epidemic progressed. At its peak, almost all patients had an adenovirus type 4 isolate (Figure 3). Isolation rates of more than 90% were seen in both male and female patients toward the end of the outbreak. At the peak of this epidemic,

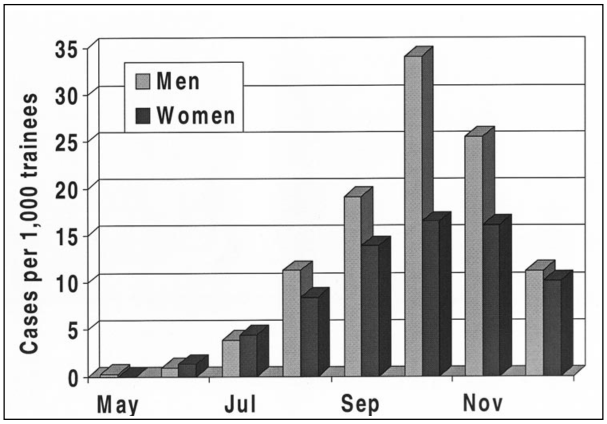

Figure 2. Patients with adenovirus type 4-associated acute respiratory disease, by sex, May–December 1997, Fort Jackson, South Carolina.

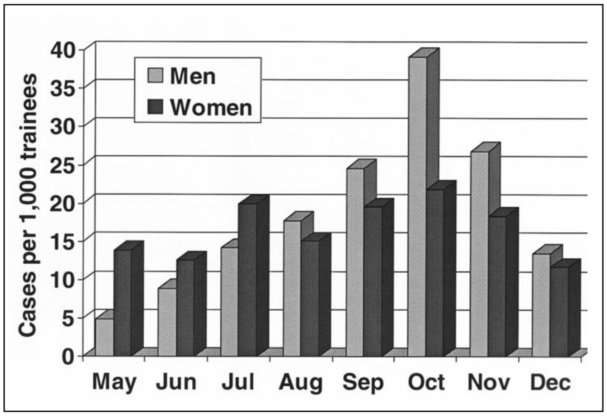

Figure 1. Patients with acute respiratory disease, by sex, May–December 1997, Fort Jackson, South Carolina.

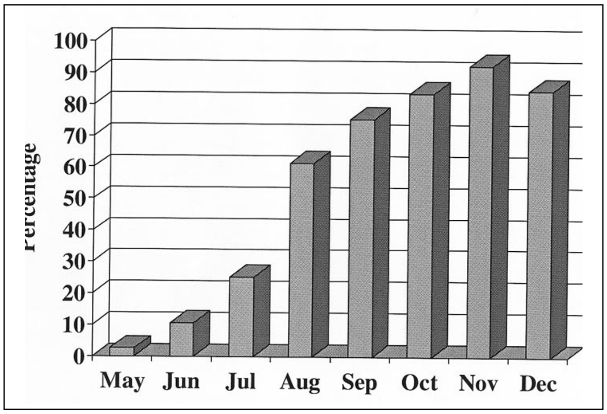

Figure 3. Percentage of total patients with acute respiratory disease who also had an adenovirus type 4 isolate, May–December, 1997, Fort Jackson, South Carolina.

approximately 70 soldiers per week were hospitalized at Fort Jackson Army Hospital. This corresponded to a weekly hospitalization rate for the entire post of approximately 1.0 admission per 100 soldiers. However, cases were not uniformly distributed throughout the trainee population but tended to occur in clusters. The outbreak intensified until November 1997, when the adenovirus vaccines were reintroduced. (Vaccination was to resume on September 1, 1997, according to the modified army policy. However, the vaccines did not reach Fort Jackson until November.) Resumption of adenovirus immunizations was associated with a rapid decline in both new cases of acute respiratory disease and isolations of adenovirus type 4. The last isolate of adenovirus type 4 was seen in December 1997. No additional isolates were reported during January through April 1998, except for a single isolate in a vaccinated trainee in February 1998. Administration of adenovirus types 4 and 7 vaccines continued through March 31, 1998.

### Conclusions

Traditionally, adenovirus vaccines were administered to male trainees entering basic combat training between October and April, but outbreaks due to types 4 and 7 occurred in the absence of vaccines. The year-round use of the two vaccines beginning in 1984 resulted in the elimination of outbreaks due to both the type 4 and type 7 viruses (3).

In 1994, administration of the vaccines was temporarily interrupted, and a limited outbreak occurred among male and female basic trainees at Fort Jackson (6). This outbreak, which began in 1995 approximately 5 to 6 weeks after the administration of the adenovirus vaccines was reinstated, was centered in one military unit that had not received the vaccines; the outbreak lasted only a few weeks, probably because the pool of susceptible soldiers was small. Concern about the threat of adenoviruses was validated by serologic data, which revealed that the adenoviral susceptibility of personnel entering the military in the 1990s was similar to that of personnel who entered in the 1970s (4).

The temporal relationship of this epidemic to suspension of adenovirus vaccination in March 1997 demonstrates the effectiveness of type-specific vaccine in controlling and preventing military epidemics. Trainees began and ended the 8-week basic combat training program each week, so it would take approximately 2 months for all trainees who received the vaccines to complete basic combat training and leave Fort Jackson. The first case of adenovirus type 4-associated acute respiratory disease occurred in May, approximately 7 weeks after vaccination was suspended. The epidemic continued until December 1997, when it was interrupted by the administration of adenovirus types 4 and 7 vaccines to all new soldiers and to all soldiers (men and women) already enrolled in basic combat training at Fort Jackson. The army hospital managed the acute respiratory disease patient workload during the 1997 epidemic, but the unexpected inpatient census proved stressful for staff and facilities.

When men and women had separate basic combat training programs, adenovirus-associated acute respiratory disease outbreaks were never documented in women, and the adenovirus vaccines were not administered to women (2,4). When the gender-integrated program started at Fort Jackson approximately 5 years ago, the decision was made to continue providing the vaccine only to men. In the limited adenovirus type 4-associated acute respiratory disease outbreak at Fort Jackson in 1995, women were hospitalized and had adenovirus type 4 isolated (6). During this large 1997 epidemic, women and men in the integrated basic combat training program were similarly affected by adenovirus type 4-associated acute respiratory disease. Early in this outbreak, admission rates for acute respiratory disease were higher in women. However, an etiologic agent was not identified. Acute respiratory disease in women in integrated basic combat training programs cannot be assumed to be the same as that observed in men and must be studied further.

Historically, adenovirus types 4 and 7 have been the major causes of acute respiratory disease in the military (2). We cannot state why type 4 caused this outbreak rather than type 7. However, documented military outbreaks demonstrate that both vaccines are necessary, since prevention of type 4-associated acute respiratory disease alone will result in the emergence of type 7-associated disease (2).

This epidemic demonstrates that adenovirus type 4 is a major threat to both male and female soldiers in basic combat training. Additionally, it confirms that large outbreaks due to adenoviruses should be expected in unvaccinated basic combat trainees.

### Acknowledgment

The authors thank Johnnie Conolly, Moncrief Army Community Hospital, Fort Jackson, SC, for her invaluable and expert technical assistance.

Dr. McNeill is a District Health Director with the South Carolina Department of Health and Environmental Control. He is a public health physician with doctoral training in Tropical Medicine. His major research interest is laboratory-based surveillance for communicable diseases.

### References

1. Foy HM. Adenoviruses. In: Evans AS, Kaslow RA, editors. Viral infections in humans. 4th ed. New York: Plenum Press; 1997. p. 119-38.
2. Gaydos CA, Gaydos JC. Adenovirus vaccines in the U.S. military. Mil Med 1995;160:300-4.
3. Brundage JF, Gunzenhauser JD, Longfield JN, Rubertone MV, Ludwig SL, Rubin FA, et al. Epidemiology and control of respiratory diseases with emphasis on group A beta-hemolytic streptococcus: a decade of U.S. Army experience. Pediatrics 1996;97:964-70.
4. Ludwig SL, Brundage JF, Kelley PW, Nang R, Towle C, Schnurr DP, et al. Prevalence of antibodies to adenovirus serotypes 4 and 7 among unimmunized U.S. Army trainees: results of a retrospective nationwide seroprevalence survey. J Infect Dis 1998;178:1776-8.
5. Hawkes RA. General principles underlying laboratory diagnosis of viral infections. In: Lennette EH, Schmidt NJ, editors. Diagnostic procedures for viral, rickettsial, and chlamydial infections. 5th ed. Washington: American Public Health Association; 1979. p. 32-5.
6. Barraza EM, Ludwig SL, Gaydos JC, Brundage JF. Reemergence of adenovirus type 4 acute respiratory disease in military trainees: report of an outbreak during a lapse in vaccination. J Infect Dis 1999;179:1531-3.

